# Impacts of Proximity to Primary Source Areas on Concentrations of POPs at Global Sampling Stations Estimated from Land Cover Information

**DOI:** 10.1021/acsomega.3c04065

**Published:** 2023-09-21

**Authors:** Jasmin K. Schuster, Tom Harner, Cassandra Rauert

**Affiliations:** † Air Quality Processes Research Section, Environment and Climate Change Canada, Toronto, Ontario M3H 5T4, Canada; ‡ Queensland Alliance for Environmental Health Sciences (QAEHS), The University of Queensland, Woolloongabba, Queensland 4102, Australia

## Abstract

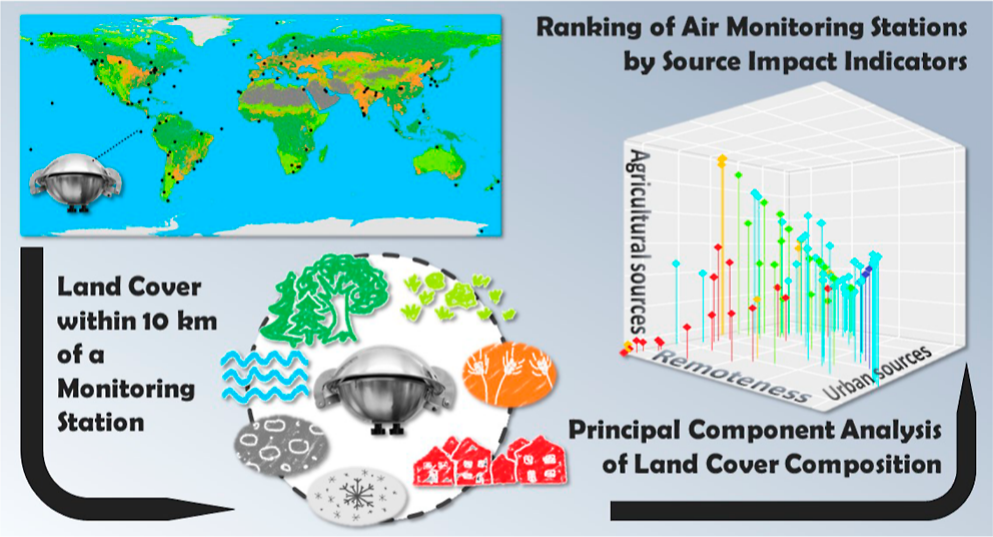

Given the considerable financial and logistical resources supporting long-term monitoring for air pollutants, and the use of these data for performance evaluation of mitigation measures, it is important to account for contributions from primary versus secondary sources. We demonstrate a simple approach for using open source Global land cover raster data from the National Mapping Organization from the Geospatial Information Authority of Japan to assess local source inputs for air measurements of legacy persistent organic pollutants (POPs)—polychlorinated biphenyls (PCBs) and organochlorine pesticides—reported under the Global atmospheric passive sampling (GAPS) Network at 119 locations for the time period 2005–2014. The land cover composition within a 10 km radius around the GAPS sites was identified to create source impact indicator (SII) vectors to quantify and rank the remoteness of the sites from human infrastructure. Using principal component analysis, three SII vectors were established to rank sites by impact of (i) general infrastructure/remoteness, (ii) urban infrastructure, and (iii) agricultural infrastructure. General infrastructure describes the combined effects of settlements and agricultural infrastructure. We found significant correlations (*p* < 0.05) between POP concentrations in air and specific SIIs. PCB levels in air had a statistically significant correlation to the SII ranking urban impacts around the sampling sites, while Endosulfan I, Endosulfan II, and Endosulfan sulfate had a statistically significant correlation with SII ranking agricultural impacts. The complete GAPS data set from 2004–2014 (1040 samples at 119 locations) was standardized based on the SII rankings to assess the global temporal trends of legacy POPs. SIIs were incorporated in the multiple regression analysis to determine global halving times. This includes short-term monitoring data from 79 locations that were previously excluded. Furthermore, the SII approach allows the integration of global monitoring data from different studies for broader global temporal trend analysis. This ability to link the results of independent and small-scale studies can enhance temporal trend analysis in support of the larger scale initiatives, such as inter alia, the Global Monitoring Plan and Effectiveness Evaluation of the Stockholm Convention in the case of POPs. This simple approach using open source data has a broad potential for application for other classes of air pollutants.

## Introduction

The Stockholm Convention on persistent organic pollutants (POPs) lists compounds that are targeted for the restriction of use and elimination from the environment. The Global Monitoring Plan (GMP) was introduced in 2005 to assess the effectiveness of the Stockholm convention through compiling data on global levels of POPs in the environment.^1^ Legacy POPs, such as polychlorinated biphenyls (PCBs) and organochlorine pesticides (OCPs), were among the original POPs listed under the convention. Despite regulation of these compounds, which in some cases predated the convention, some POPs continue to exhibit enduring high concentrations in air and slowly declining levels. An ongoing question, related to the effectiveness evaluation of the convention, is the extent to which primary sources (i.e., old stocks, landfills) and secondary sources (e.g., revolatilization from other environmental compartments such as soil, water) affect the temporal trends in levels observed in air.^2^

The Global Atmospheric Passive Sampling (GAPS) network was established to provide information on spatial and temporal trends of POP levels in air to the GMP^3^ (Figure S1a). The GAPS data inform the effectiveness evaluation of the convention and also yield invaluable information for understanding long-range transport in air. The site categories used under the GAPS network (“Urban”, “Agricultural”, “Rural”, “Background”, “Polar”) are a streamlined version of the categories set down in the guidance document on the GMP for POPs^4^ [Site type: urban, suburban, rural, remote, high altitude, polar, marine/coastal; potential source type: industrial, traffic, residential, agricultural, waste sector, none (i.e., continental background site), <10 km]. Considering urban and agricultural sources as the main driver, streamlining the site categories allows for a concise overview of the global range of POP concentrations. Since the start of the GAPS network in 2005, it became clear that localized sources and conditions impacted POP concentrations at some sites (Figure S1b,c). POP concentrations monitored at some “background” sites were found to be on the same order of magnitude as concentrations reported for “agricultural” and “urban” sites (Figure S1b,c). This indicated the presence of important and broad-scale sources that were not captured in the previous site classification based on the assessment by our individual local contacts.

Several sophisticated models address the global emission and transport of POPs based on source areas, such as the remoteness index,^5^ transfer efficiency,^6^ or the pertingency index.^7^ However, due to their scope, the grid sizes of these models range from 4 × 5°, 3.75 × 3.75°, and 1 × 1°, respectively, which means that the smallest cell covers an area of about 111 × 111 km. While this provides valid information on the global POP distribution, the resolution is not sufficient to capture local sources, which affect POP concentrations in air around a monitoring site. In addition, air parcel back-trajectory analysis, which is suitable for identifying potential general source regions, is less useful for evaluating specific local sources.^8^

**Figure 1:**
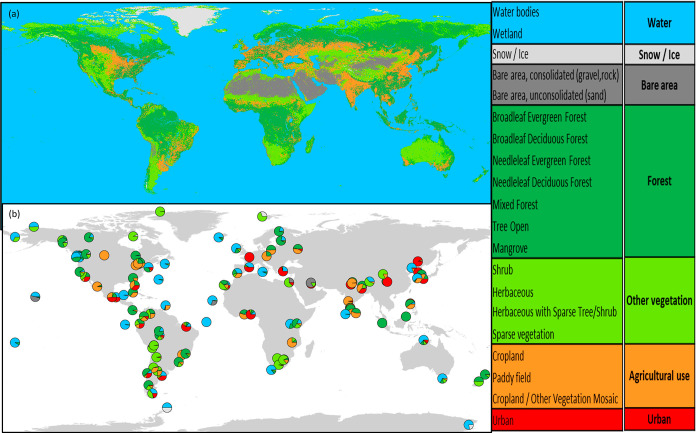
GLC raster files from the NMO with 15 arcseconds resolution are plotted on the world map (a). The land cover composition within a 10 km radius around each of the 119 GAPS sites was determined (b).

The National Mapping Organization (NMO) from the Geospatial Information Authority of Japan provides a Global land cover (GLC) raster^9^ based on moderate resolution imaging spectroradiometer (MODIS) data with 500 m resolution of 20 different land cover types (Figure 1). The higher resolution of the GLCNMO data allows the identification of possible source areas (i.e., urban, cropland, paddy field) around monitoring stations.

This study introduces source impact indicators (SIIs) as a simple tool to standardize and rank different monitoring stations by land use, which allows improved assessment of possible source impacts. Principal component analysis (PCA) was used as a ranking tool^10–12^ to transform the land cover information within a 10 km radius around the monitoring stations to the SII vectors. The GAPS network provides a suitable data set to evaluate the SIIs. SIIs were established for 119 GAPS monitoring stations, capturing the impact of (i) general infrastructure/remoteness, (ii) urban infrastructure, and (iii) agricultural infrastructure. General infrastructure describes the combined effects of settlements and agricultural infrastructure. It encompasses primary sources as well as the potential of human activity to facilitate the reemission from secondary sources. The SIIs were then applied to PCB and OCP data collected from these stations to establish statistically significant correlations between potential local source impacts (i.e., cropland, urban infrastructure) and monitored POP concentrations in air. Temporal trends were previously reported from continuous measurements at a subset of 40 sites under the GAPS network (including 66% of the total reported data from the network and 7–26 samples per site). Small data sets at individual sites pose a challenge to statistically robust temporal trend analysis. A global temporal trend analysis was performed on the PCB and OCP data from all 119 sites (*n* = 1040 samples) from the GAPS network for 2004–2014.^13^ The data from all sites, including those with only short-term measurements (*n* = 350 samples), were standardized based on SII vectors. Multiple linear regressions were applied to establish robust global temporal trends. This approach reduced the impact of data outliers on statistical analysis.

The approach demonstrated here for long-term POPs monitoring under the GAPS Network can be extended to other air pollutant classes, where it is important to distinguish local/primary versus secondary source inputs and to integrate among monitoring programs.

## Methods

### Assessment of Land Cover

The land cover surrounding the individual sampling sites within a 10 km radius was identified and quantified based on GLC raster files from the NMOs^9^ from the Geospatial Information Authority of Japan (GLCNMO Version 3, combined MODIS data from Terra and Aqua 2013, WGS84, 15 arcseconds resolution). The GLCNMO data differentiates 20 land cover categories: (1) broadleaf evergreen forest, (2) broadleaf deciduous forest, (3) needleleaf evergreen forest, (4) needleleaf deciduous forest, (5) mixed forest, (6) tree open, (7) shrub, (8) herbaceous, (9) herbaceous with sparse tree/shrub, (10) sparse vegetation, (11) cropland, (12) paddy field, (13) cropland/other vegetation mosaic, (14) mangrove, (15) wetland, (16) bare area, consolidated (gravel, rock), (17) bare area, unconsolidated (sand), (18) urban, (19) snow/ice, and (20) waterbodies. Previous studies by Choi et al. (2008) in remote areas have shown a POP concentration decline in air within 1 km of a local point source.^14^ A recent passive air sampling campaign in the Athabasca oil sands region, an area defined by broader-scale localized sources, showed increased pollutant levels in air within 5–10 km of an emerging local point source, while levels at higher distances remained consistent.^15^ A radius of 10 km was chosen here to capture the impact of localized sources as well as to describe the general infrastructure and remoteness of the sampling region.

ESRI ArcMap 10.6.1. with spatial analyst was used to extract the required land cover information from the grid data and transform it to numerical area data expressed as square meters (*m*^2^). Details on the method and tools are reported in the Supporting Information. The 20 land cover categories were further reduced to seven consolidated categories that share major characteristics impacting the release/removal of POPs in the environment [waterbodies (wetland, water bodies), forest (broadleaf evergreen forest, broadleaf deciduous forest, needleleaf evergreen forest, needleleaf deciduous forest, mixed forest, tree open, mangrove), open vegetation (shrub, herbaceous, herbaceous with sparse tree/shrub, sparse vegetation), bare area bare area, consolidated (gravel, rock), bare area, unconsolidated (sand)], snow/ice, cropland, urban) (Figure 1a). The land composition for the 119 GAPS sites is visualized in Figure 1b and listed in the Excel Supporting Information Table S2.

### Ranking of GAPS Sites Using PCA

PCA has been applied as a ranking tool in different fields^10–12^ with the aim to reduce the dimensionality of characteristic parameters for the ranking subjects. The goal for this study is to rank 119 individual GAPS sites based on general impacts of infrastructure/remoteness and the type of infrastructure. PCA was performed in the open source software R 4.1.0^16^ using the standard function “prcomp” on the grouped land cover parameters “water”, “land” (= snow/ice, bare area, forest, open vegetation, agricultural, urban), “no infrastructure” (= water, snow/ice, bare area, forest, open vegetation), “agricultural”, and “urban”. The resulting principal components (PC) were applied as SII.

### Legacy POP Data from the GAPS Network

Passive air sampler-derived concentrations of legacy POPs are available for 2005–2014 for 119 sites under the GAPS network.^13^ Continuous long-term data during this period are available from 40 sites. Each sampling year has an average of 56–60 operating sites. Concentrations have been reported from these sites for the sum of seven PCBs (∑_7_PCBs) (PCB 28, PCB 52, PCB 101, PCB 118, PCB 138, PCB 153, PCB180), Endosulfan I/II/sulfate (SO_4_), α-/γ-hexachlorocyclohexane (HCH), *cis*-/*trans*-chlordane, *trans*-nonachlor, heptachlor, heptachlor epoxide, and dieldrin as integrated concentrations for consecutive three-month deployment periods. Data are available for 2005–2007, 2009, 2011, and 2014 (1040 samples in total). Details on sampling procedure and data treatment are reported elsewhere.^13^

Multiple linear regression was performed in R 4.1.0 for the geometric mean concentrations (logarithm applied) at each GAPS site with the SII1, SII2, and SII3 to assess correlations between levels in air and source impacts. Furthermore, multiple linear regression was performed on all available data points for the concentrations [natural logarithm (ln) applied] at each GAPS site with the median dates of the sampling periods, SII1, SII2, and SII3 to assess global temporal trends. The temporal trends in air, defined by halving/doubling times, were estimated from slopes following first order kinetics^13^ (i.e., halving/doubling time are estimated by dividing ln2 by the slope values for the temporal component of the multiple regression). Value ranges for the halving and doubling times are based on the reported uncertainties of the R model. The halving/doubling times are compared to previously reported values by Schuster et al. 2021.^13^

## Results and Discussion

### Updating Sampling Site Location-Based SIIs

The GAPS sites are classified as “polar”, “background”, “rural”, “agricultural”, and “urban”. The GAPS network, as with many other networks that operate sampling sites remotely, relies heavily on partnerships with local contacts for deploying samplers. Other classification information is often provided by these site contacts, including the approximate distances from settlements and possible sources of POPs, the surrounding landscape, and the extent of human activity near the site. This information is very valuable, but the input and interpretation can be biased based on the assessor. Using GLCNMO data to characterize the sites reduces this input bias.

**Figure 2:**
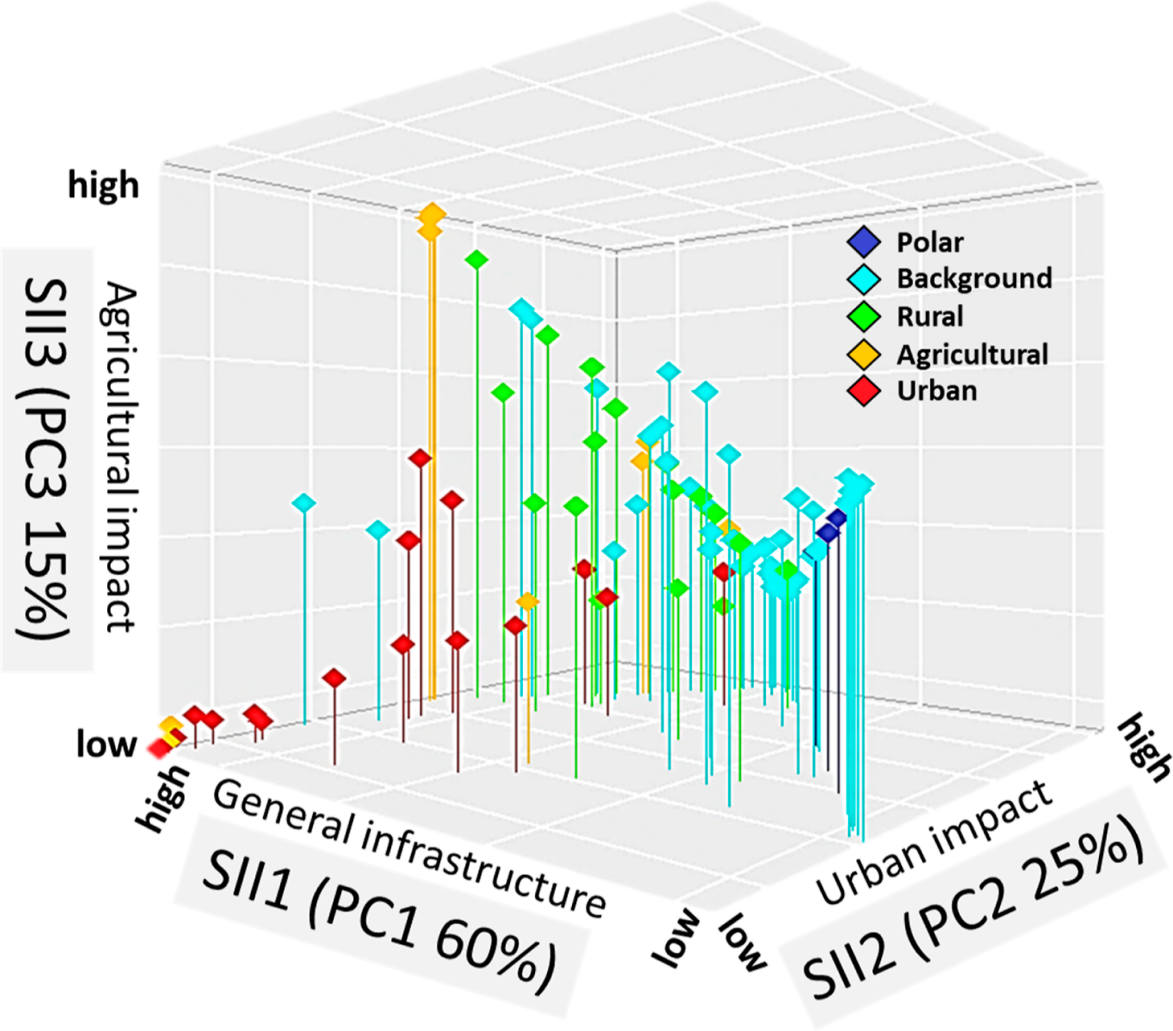
SIII for 119 GAPS sites is plotted to illustrate the impacts of total infrastructure and infrastructure type. The site types assigned under the GAPS network are identified by color.

The GLCNMO data are required to provide information on general remoteness and the local impacts of urban and agricultural sources on individual GAPS sites. PCA was performed on 119 GAPS sites based on the land cover factors “no infrastructure”, “urban”, “agricultural”, “land”, and “water” and resulted in 5 PCs, of which the first three explained 99% of the variance of components (Table 1) and were selected for interpretation. PC1 explained 59% of the variance and showed strong correlation with decreasing presence of infrastructure, while PC2 (25%) and PC3 (15%) showed strong correlation with the factors “urban” and “agricultural”, respectively. Going forward, the PC scores of PC1, PC2, and PC3 are referred to as SIIs with SII1 (PC1) ranking the general impact of infrastructure or remoteness, SII2 (PC2) the impact of urban infrastructure, and SII3 (PC3) the impact of agricultural infrastructure (i.e., cropland, paddy fields) in each GAPS site. The histograms for the SIIs (Figures S2) show that most GAPS sites fall in the lower to mid range of possible source impacts. Figure 2 visualizes the placement of the GAPS sites along the SII rankings. The majority of sites that were originally classified as “urban” and “agricultural” under the GAPS network showed a similar classification based on their SII rankings. These sites are typically in areas with higher infrastructure and would, therefore, have been fairly well characterized by site operators using previous narrative methods. An interesting outcome was that some “background” and “rural” sites also ranked high in the SII scales, which reveals urban and agricultural impacts that might not have been considered in previous characterization and data interpretation from these locations. Table 2 shows the percentile rank ranges for each site type in the three SII scales with a wide variance for all five GAPS site classifications (i.e., a percentile rank range of 0–92% for the “background” sites). The additional classification of sites based on SIIs narrows the characterization of a location type by assigning numerical values. This further allows a wider range of statistical interpretation of the monitored concentrations in air such as multiple linear regression of concentrations in air with SIIs.

**Table 1:** PCA on the Factors “No Infrastructure”, “Urban”, Agricultural”, “Land”, “Water” Lead to Three PCs for the Ranking of the GAPS Sites^a^

PCA results	PC1	PC2	PC3
standard deviation	0.52	0.34	0.27
proportion of variance [%]	59	25	16
cumulative proportion [%]	59	84	100

correlation	PC1	PC2	PC3
no infrastructure	*0.79	0.57	–0.20
urban	–0.51	*–0.70	–0.50
agricultural	–0.52	–0.01	*0.85
land	–0.89	0.44	–0.14
water	0.89	–0.44	0.14

a Based on the correlation between PC scores and the land cover factors, the SIIs were assigned as PC1 = SII1 no infrastructure/remoteness, PC2 = SII2 urban, and PC3 = SII3 agricultural

**Table 2:** SII Percentile Ranks for the Different GAPS Site Types “Polar” (*n* = 5), “Background” (*n* = 68), “Rural” (*n* = 18), “Agricultural” (*n* = 10), and “Urban” (*n* = 18) Show a Wide Variance and Indicate That an Additional Classification System is Beneficial for Data Interpretation

percentile ranking for GAPS site classification	SII1 (%)	SII2 (%)	SII3 (%)
polar	9–44	0–69	11–63
background	0–92	10–89	13–95
rural	15–88	25–87	10–96
agricultural	34–97	22–97	2–100
urban	31–100	36–100	0–82

### Analyzing Global Concentrations of Legacy POPs in Air Based on SIIs

The GAPS network has reported legacy POP concentrations in air for 2005–2014 at 119 locations. We differentiate between POPs applied in industrial and urban settings such as PCBs and those applied as pesticides in agricultural and urban settings (Endosulfan I, Endosulfan II, Endosulfan SO4, γ-HCH, α-HCH, *cis*-chlordane, *trans*-chlordane, *trans*-nonachlor, heptachlor, heptachlor epoxide, dieldrin). While these compounds have been regulated and listed under the Stockholm convention for decades, the atmospheric concentrations of many are still controlled by primary source emissions,^13,17^ though the impact of secondary sources is becoming more prevalent.^2^ For chemicals with ongoing primary source emissions, the concentration gradient between near source sites toward sites in remoter areas is expected to be a steeper decline when compared to the concentration gradients of POPs with dominant secondary source emissions. The increasing impact of POP emissions from secondary sources could reduce this gradient, i.e., the correlation between SII vectors and POP concentration in air.

**Figure 3:**
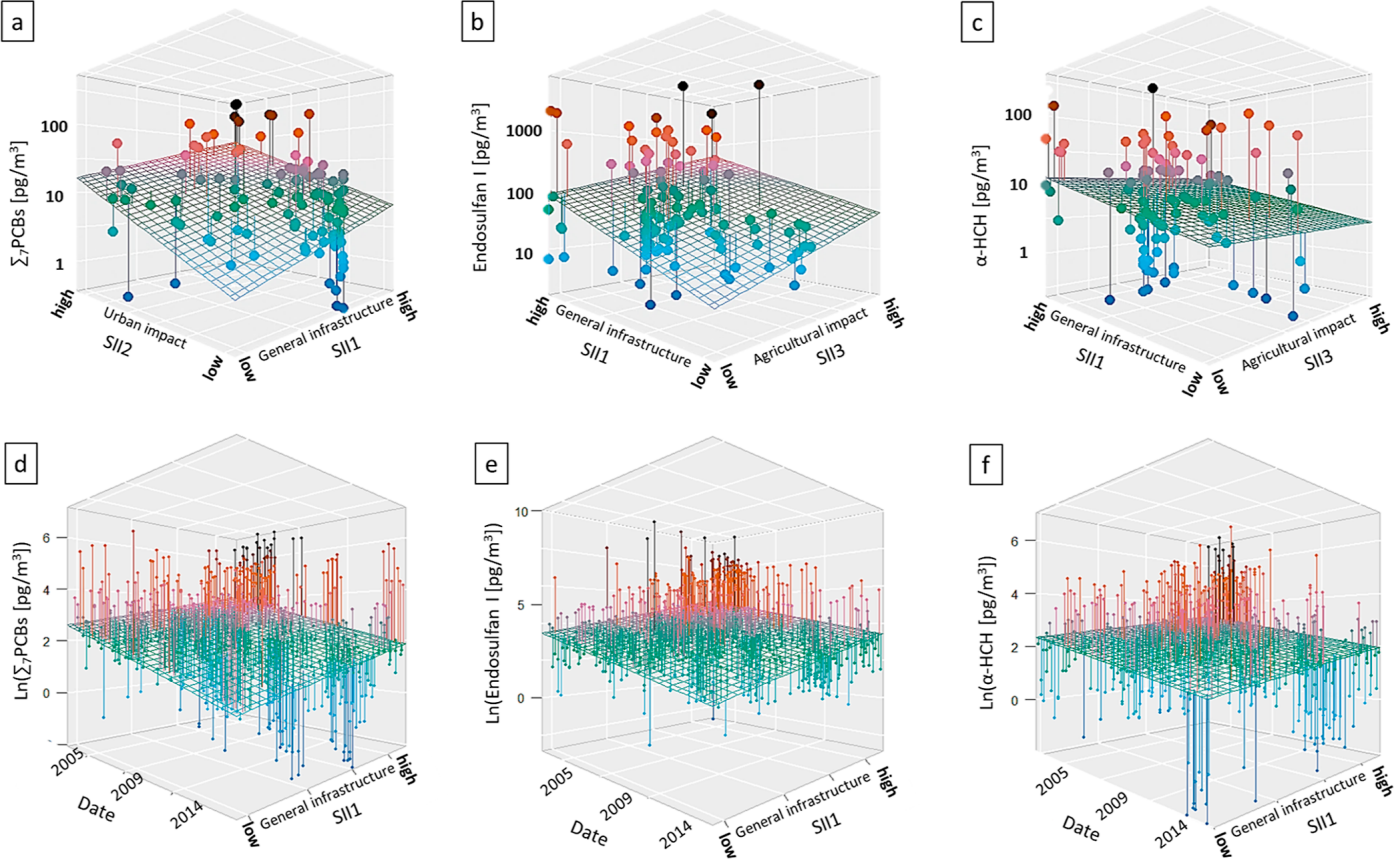
Multiple linear regression analysis with the SIIs showed statistically significant correlation for ∑_7_PCBs with SII1 and SII2 (a), as well as Endosulfan I with SII1 and SII3 (b). The correlation of α-HCH with the SIIs was significantly weaker (c). Similarly, when establishing global temporal trends, the correlations for ∑_7_PCBs (d) and Endosulfan I (e) were stronger than for α-HCH (f). Correlation in the graphs is visualized with a regression plane.

Multiple linear regression analysis was performed for the set of legacy POPs and SIIs (Table S1). The results show overall a statistically significant correlation with SII1 (general infrastructure/remoteness), *p* < 0.05, while the correlation with SII2 (urban impact indicator) and SII3 (agricultural impact indicator) varied depending on the chemical (Figure 3a–c). ∑_7_PCBs showed a significant correlation with SII1 and SII2 (Figure 3a). As chemicals with mostly urban and industrial applications, the statistically significant correlations with the SIIs (*p* = 4 × 10^–04^ and 7 × 10^–09^, respectively) for general infrastructure and urban impact is to be expected, especially if PCB concentrations in air are still mainly driven by primary source emissions. Compared to that, atmospheric concentrations of Endosulfan I/II and its degradation product Endosulfan SO4 show statistically significant correlations with SII1 and SII3 (*p* = 3 × 10^–05^ and 1 × 10^–02^, respectively, Figure 3b). Endosulfan I/II is a pesticide with recent applications mostly in the agricultural sector. This is reflected in the strong correlation with the SII for the agricultural impact. Endosulfan concentrations in air are still driven by primary source emissions. α-HCH and γ-HCH show a less pronounced relation to the SIIs. While there is a statistically significant correlation for SII1, there is no significant correlation for SII3 (Figure 3c). This could be due to increasing reemissions of HCHs from secondary sources buffering the concentration gradient between near source and remote locations.^2^*cis*-chlordane, *trans*-chlordane, *trans*-nonachlor, and heptachlor show only a correlation to the urban SII2. Chlordane and heptachlor were predominantly used for ant and termite control, landscaping with limited agricultural applications.^18^ The lack of correlation between concentrations in air and the general impact of infrastructure (SII1) could also indicate an increasing impact of secondary sources for this chemical group. Dieldrin showed a statistically significant correlation with all SIIs. This reflects the use of dieldrin in both the agricultural sector and urban settings (i.e., wood treatment against termites, cloth treatment against moths).^19–21^ The presented correlations between the SII factors for the results from the multiple linear regression analysis show that there are significant correlations between local POP concentrations and SIIs. The land use compilation for identifying the SIIs has been shown to be a valid approach to identify and rank the general impact of proximate infrastructure on the POP concentrations in air, as well as more specific sources such as urban or agricultural infrastructures.

The SII vectors are developed as a ranking tool based on high resolution local data at this step. Future work would connect this approach with global large scale data such as global production/emission inventories, global night light data, directional and Hysplit modeling, or outputs from models that already employ these factors, such as the remoteness index,^5^ transfer efficiency,^6^ or the pertingency index.^7^

### Alternative Method to Assess Global Temporal Trends

The GAPS network has reported data from 119 locations for the years 2005–2007, 2009, 2011, and 2014, which makes up a total of 1040 air samples.^13^ However, continuous data are only available from 40 of those sampling sites, which limited the determination of temporal trends to incorporate only 66% of the available data. The application of the SIIs in multiple linear regression analysis for first order kinetics allows integrating the complete POP data set in the temporal trend analysis (Figure 3d–f) by assigning single data point sites a ranking in the multidimensional system in relation to other sampling sites. This is a major advancement in the approach for compiling data for the purpose of trend assessment as it allows for inclusion of data from multiple locations, where some stations may have operated intermittently or for only short periods of time. Given the high costs and effort associated with undertaking air measurements of POPs, this new approach will help to make best use of available monitoring data. This could potentially lead to enhanced reporting under the GMP and more inclusive, integrated, and cost-effective approaches to support effectiveness evaluation of the Stockholm convention on POPs.

**Figure 4:**
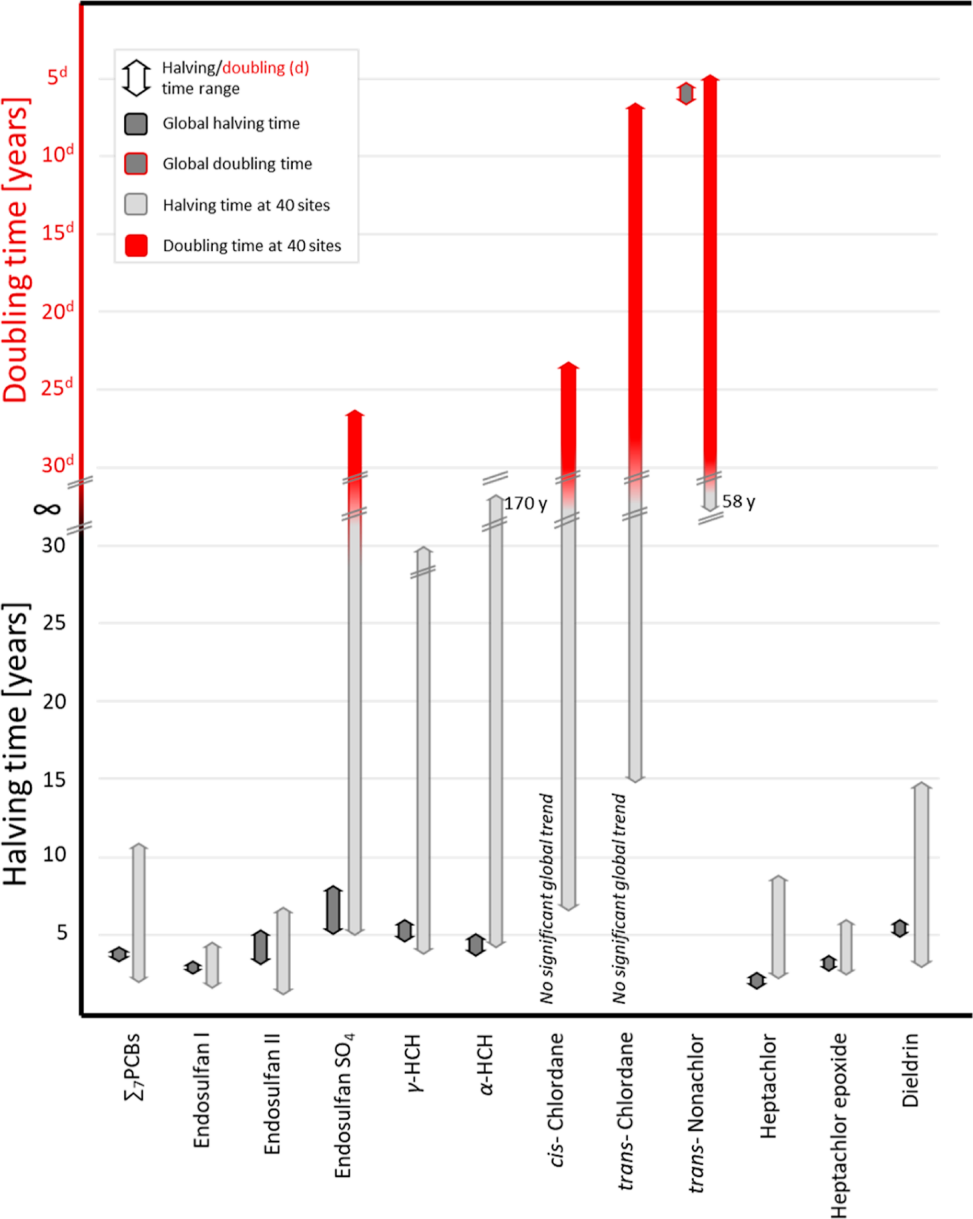
Global temporal trend slopes estimated from 1040 samples between 2005 and 2014 correspond to temporal trend slopes estimated for 40 individual GAPS sites (66% of data, 7–26 samples per site). Statistical details are presented in Supporting Information Table S1.

The results of the temporal trend analysis based on the SII approach were statistically significant for all compounds with the exception of the chlordanes. This further suggests an increasing impact of secondary sources on chlordane concentrations in air buffering primary source signals. Similar to this, *trans*-nonachlor showed a global increase over time (doubling time 5.2–6.9 years). The global halving times of α-HCH and γ-HCH show a significant decline over time, whereas the temporal trends observed at individual sites indicate a transition state to a more pronounced impact of secondary sources. The global halving times of the other analytes (Figure 4) are in the same order of magnitude as the halving times *t*_hi_ established for individual sites (Table S1 with 3.5–4.1 years for ∑_7_PCBs (*t*_hi_ = 2.3–11 years), 2.8–3.3 years for Endosulfan I (*t*_hi_ = 2.1–4.6 years), 3.7–5.2 years for Endosulfan II (*t*_hi_ = 1.6–6.7 years), 5.1–7.9 years for Endosulfan SO_4_ (*t*_hi_ = 5.0-doubling 27 years), 4.1–5.1 years for α-HCH (*t*_*hi*_ = 4.4–170 years), 4.8–6.2 years for γ-HCH (*t*_hi_ = 4.1–30 years), 1.9–2.4 years for Heptachlor (*t*_hi_ = 2.2–8.0 years), 3.1–3.7 years for Heptachlor epoxide (*t*_hi_ = 2.9–5.8 years), and 5.0–6.2 years for dieldrin (*t*_hi_ = 3.3–14 years). These values are overall in line with those reported by established monitoring networks such as the Great Lakes Integrated Atmospheric Deposition Network,^22,23^ the Arctic Monitoring and Assessment Programme,^24^ the European Monitoring and Evaluation Programme,^25^ the MONnitoring NETwork MONET,^17^ and the Toxic Organic Micro Pollutants network.^26^ The uncertainty and range observed for the global halving times are significantly smaller than for the range observed for the halving times over the different individual sites. The temporal trend assessment at individual sites is based on significantly smaller data sets and is affected more by outliers and data levels close to detection limits than the global temporal trend assessment. Differing temporal trends at individual sites are not captured under this method and should still be determined when sufficient data are available. Establishing global halving time, as shown here, is a robust approach for big data sets over multiple years that lack consistent data at individual sites and consolidating data from multiple studies. In future work, it may be possible to extend this approach to compile and analyze air monitoring data from different sources and programs, accounting for comparability issues among programs, in order to derive global trends. Future work can also include an assessment of how to further refine land-use data, e.g., by differentiating forest types (coniferous vs deciduous) that may impact chemical deposition and revolatilization and concentration in air. This new direction in trends assessment is cost-and resource-effective. It will help to integrate data from different sources in order to improve reporting capability as many programs face growing challenges associated with measuring a growing list of POPs and other pollutants in air.^27^ The GIS-based normalization of sampling sites developed here has promise as a tool for optimizing data compilation and analysis as part of global scale assessments, where many sites and programs are involved,^17,24,28–31^ such as, inter alia, under the GMP of the Stockholm convention on POPs.

## Supporting Information Available

Map of GAPS sites with classification; table with statistical parameters; and method for extracting land cover information (PDF)
Table with site information and land cover details (XLSX)
