# Supplementary material for: Impacts of Proximity to Primary Source Areas on Concentrations of POPs at Global Sampling Stations Estimated from Land Cover Information
Source: ACS Omega. 2023 Sep 21;8(39):36016–24. doi: 10.1021/acsomega.3c04065 (PMC10552113; doi:10.1021/acsomega.3c04065)
Supplement: Supplementary file 1 — ao3c04065_si_001.pdf [file ao3c04065_si_001.pdf]

Impacts of proximity to primary source areas on concentrations of POPs at global sampling stations estimated from land cover information

Jasmin K. Schuster[1]*, Tom Harner[1], Cassandra Rauert[1,2]

[1]Air Quality Processes Research Section, Environment and Climate Change Canada, Toronto, Ontario, M3H 5T4. Canada

[2]Queensland Alliance for Environmental Health Sciences (QAEHS), The University of Queensland, Woolloongabba, Queensland, 4102, Australia

E-mail contact: jasmin.schuster@ec.gc.ca

Contents

Figure S1. Map of GAPS sites with site classification 1

Figure S2. Histograms for the distribution of GAPS sites across SII scores 2

Table S1. Overview of statistical parameters for multiple linear regression of SIIs and concentrations in air 3

Text S1. Extracting Land cover information from ESRI ArcMap 10.6.1. with Spatial Analyst 4

Figure S1. Map of GAPS sites with site classification

Between 2005-2014 a total of 119 GAPS sites was established (a). GAPS sites are assigned a site type based on locations and communications with local contacts (“polar”, “background”, “rural”, “agricultural”, “urban”). The reported air concentrations for legacy POPs at these sites [1] show wide ranges within their assigned site type, especially for “background” sites. Some background site concentrations of $\sum_7$PCBs are the same order of magnitude as data reported from “urban” sites (b). Endosulfan I data from “background” sites is the same order of magnitude as reported at “agricultural” sites (c).

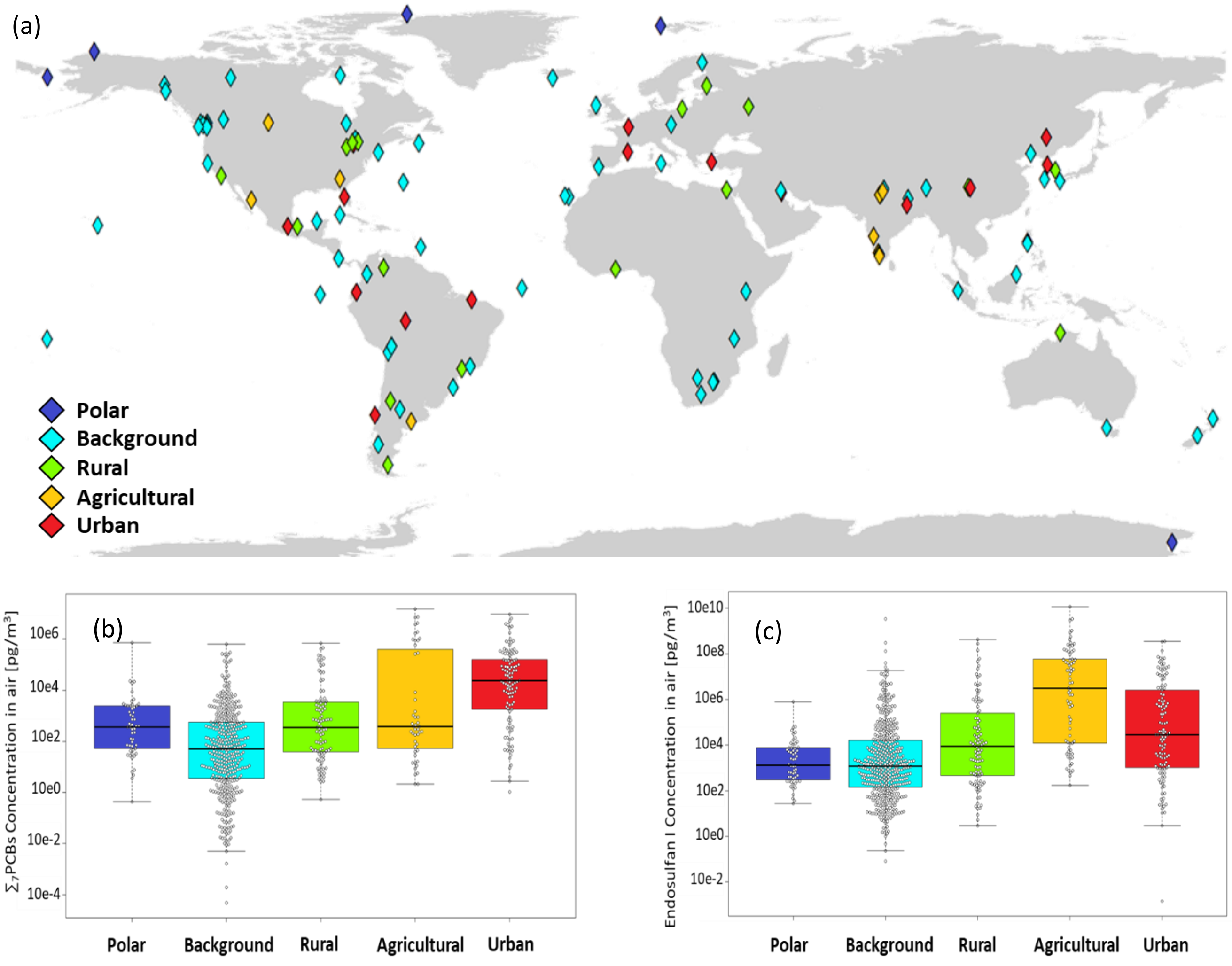

Figure S2. Histograms for the distribution of GAPS sites across SII scores

Histograms show a higher frequency of GAPS sites along the SII scores at the lower to mid range of the SII score.

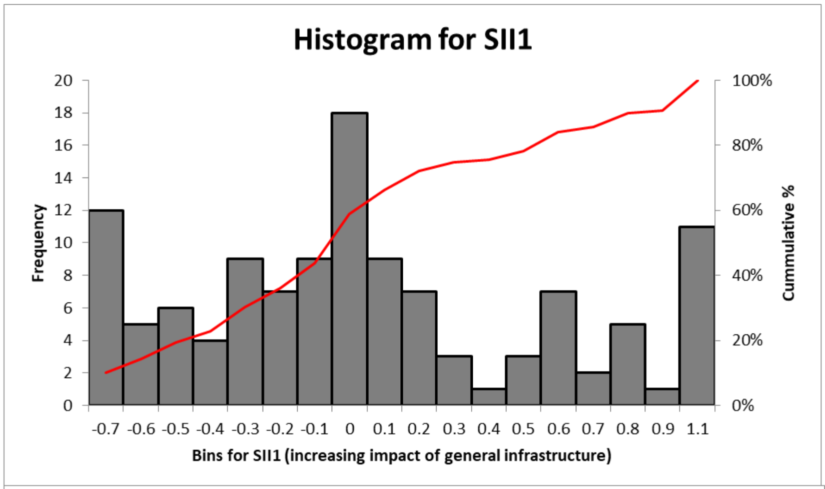

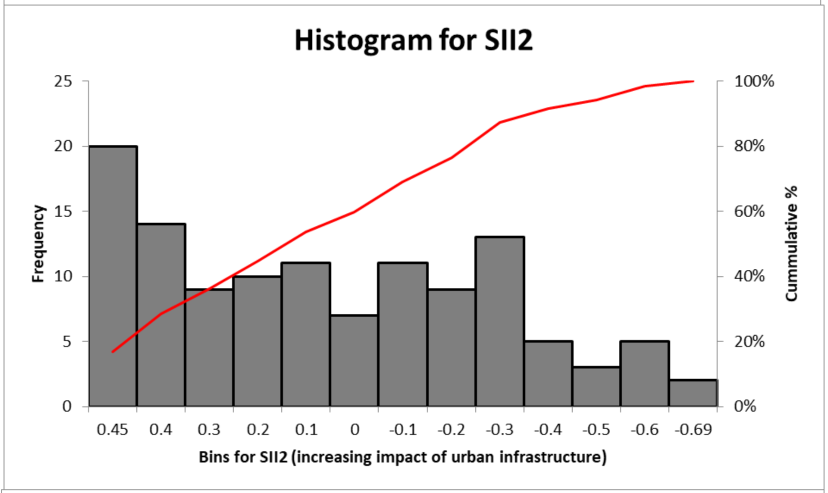

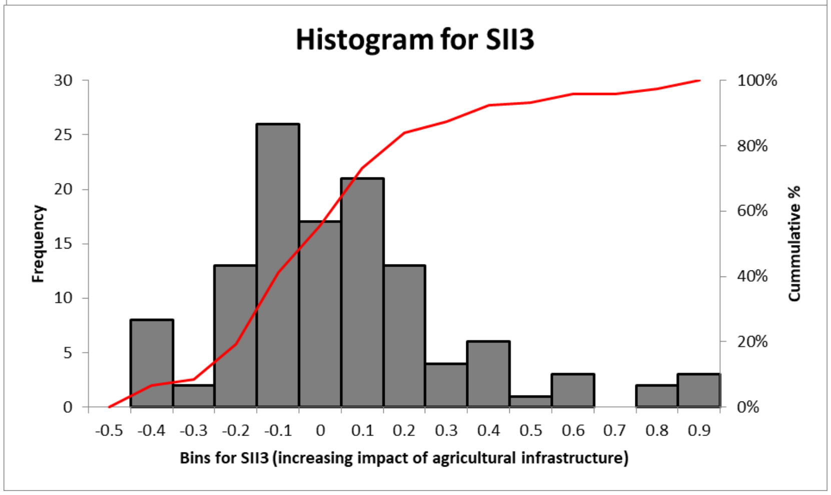

Table S1. Overview of statistical parameters for multiple linear regression of SIIs and concentrations in air

Multiple linear regression analysis was performed for the geographic mean concentrations at all GAPS sites between 2005-2014 and the SIIs. Furthermore, multiple linear regression analysis was performed for the complete air concentration data (natural logarithm) for correlation with the average sampling dates and SIIs to establish global halving (doubling) times based in first order kinetics. Halving (doubling) times estimated from air concentrations at individual GAPS sites was previously reported by Schuster et al. (2021) [1]and is reported below for comparison.

Factors	Statistics	Σ_7PCBs	Endosulfan I	Endosulfan II	Endosulfan SO_4	γ-HCH	α-HCH	*cis*-Chlordane	*trans*-Chlordane	*trans*-Nonachlor	Heptachlor	Heptachlor epoxide	Dieldrin
all factors	p-value	1.E-09	5.E-06	5.E-06	6.E-06	5.E-07	3.E-02	7.E-04	2.E-04	4.E-03	2.E-03	8.E-02	2.E-07
	R^2	0.36	0.23	0.25	0.24	0.27	0.08	0.17	0.19	0.14	0.25	0.09	0.33
SII1	Slope	-0.91	-1.36	-1.38	-1.40	-1.25	-0.78	-0.37	-0.49	-0.27	-0.94	-0.68	-0.76
	Error	0.25	0.31	0.37	0.32	0.27	0.33	0.25	0.27	0.26	0.49	0.37	0.21
	p-value	4.E-04	3.E-05	3.E-04	3.E-05	1.E-05	2.E-02	1.E-01	8.E-02	3.E-01	6.E-02	7.E-02	6.E-04
SII2	Slope	-2.29	-0.90	-1.65	-0.65	-1.40	-0.72	-1.36	-1.64	-1.27	-2.43	-0.88	-1.57
	Error	0.36	0.46	0.55	0.46	0.39	0.46	0.34	0.39	0.38	0.75	0.53	0.32
	p-value	7.E-09	5.E-02	3.E-03	2.E-01	5.E-04	1.E-01	2.E-04	6.E-05	1.E-03	2.E-03	1.E-01	5.E-06
SII3	Slope	0.00	1.49	1.70	1.53	0.19	0.06	0.27	0.37	0.65	-1.17	-0.62	0.91
	Error	0.48	0.57	0.67	0.56	0.48	0.58	0.47	0.51	0.48	0.93	0.68	0.40
	p-value	1.E+00	1.E-02	1.E-02	8.E-03	7.E-01	9.E-01	6.E-01	5.E-01	2.E-01	2.E-01	4.E-01	3.E-02
Temporal trend	Slope	-5.E-04	-6.E-04	-4.E-04	-3.E-04	-3.E-04	-4.E-04	7.E-05	8.E-06	3.E-04	-9.E-04	-6.E-04	-3.E-04
	Error	4.E-05	4.E-05	8.E-05	7.E-05	4.E-05	5.E-05	4.E-05	5.E-05	5.E-05	1.E-04	5.E-05	4.E-05
	p-value	<2.E-16	<2.E-16	1.E-08	6.E-06	1.E-15	<2.E-16	1.E-01	9.E-01	4.E-12	1.E-14	<2.E-16	<2.E-16
global	halving/doubling time [y]	3.5 - 4.1	2.8 - 3.3	3.7 - 5.2	5.1 - 7.9	4.8 - 6.2	4.1 - 5.1	na	na	5.2[d] - 6.9[d]	1.9 - 2.4	3.1 - 3.7	5.0 - 6.2
individual sites	halving/doubling time [y]	2.3 – 11	2.1 – 4.6	1.6 – 6.7	5.0 – ***27***[d]	4.1 - 30	4.4 - 170	5.9 – ***23***[d]	15 – ***7***[d]	58 – ***4.9***[d]	2.2 – 8.0	2.9 – 5.8	3.3 - 14

Text S1. Extracting Land cover information from ESRI ArcMap 10.6.1. with Spatial Analyst

The Global Land Cover by National Mapping Organizations (GLCNMO) is geospatial information in raster format which classifies the status of land cover of the whole globe into 20 categories. The data is available in GeoTiff format (4 tiles at 90 degrees in latitude and 180 degrees in longitude, or 21,600 pixels by 43,200 pixels with 15 second resolution, WGS84).

Spatial Analyst and the basic tools available in ArcToolbox are used in ArcMap to transform and extract the land cover data.

The Raster data was first converted to Polygon data (ArcToolbox > Conversion Tools > From Raster > Raster to Polygon, do not simplify polygons) and then merged for the four tiles (ArcToolbox > Data Management Tools > General > Merge). The area around each sampling site was assigned a buffer with 10 km radius (ArcToolbox > Analysis Tools > Proximity > Buffer). The buffer was used to clip the landcover features within a 10 km radius of the sampling sites (ArcToolbox > Analysis Tools > Extract > Clip). The resulting files contain polygon information with area and land use code. Features that are connected over multiple polygons have to be split into separate polygons (ArcToolbox > Data Management Tools > Features > Multipart to Singlepart). Next, the sampling site IDs are assigned to their assorted polygons (ArcToolbox > Analysis Tools > Overlay > Spatial Join). In the Attribute table of the final layer, containing the polygons for the landcover associated with the sampling site IDs, the polygon areas are estimated. The table is extracted for further processing in Excel (ArcToolbox > Conversion Tools > Excel > Table to Excel).

The assembled data for land cover and SII vectors is available in the Excel SI file.

1. Schuster, J.K., T. Harner, A. Eng, C. Rauert, K. Su, K.C. Hornbuckle, and C.W. Johnson, *Tracking POPs in Global Air from the First 10 Years of the GAPS Network (2005 to 2014).* Environmental Science & Technology, 2021.
